# Cardiac and hepatic siderosis in myelodysplastic syndrome, thalassemia and diverse causes of transfusion-dependent anemia: the TIMES study

**DOI:** 10.1097/HS9.0000000000000224

**Published:** 2019-06-01

**Authors:** P. Joy Ho, Devendra Hiwase, Raj Ramakrishna, Nicholas Viiala, Ann Solterbeck, Robert Traficante, Evren Zor, Othon L. Gervasio, Laura M. High, David M. Ross, Donald K. Bowden

**Affiliations:** 1 Royal Prince Alfred Hospital, Sydney, Australia; 2 Sydney Medical School, University of Sydney, Sydney, Australia; 3 Haematology, Royal Adelaide Hospital, Adelaide, Australia; 4 Southern Sydney Haematology, Kogarah, Sydney, Australia; 5 Liverpool Hospital, Sydney, Australia; 6 Statistical Revelations, Melbourne, Australia; 7 Novartis Pharmaceuticals, Sydney, Australia; 8 Flinders University and Medical Centre, Adelaide, Australia; 9 Monash Medical Centre, Melbourne, Australia.

## Abstract

The significant morbidity and mortality associated with iron overload can be reduced by effective iron chelation. Magnetic resonance imaging (MRI) provides accurate and reproducible iron load assessment. The aim of this epidemiological study was to assess the prevalence and severity of cardiac and hepatic siderosis by MRI and to evaluate the impact of MRI on clinical management in patients with transfusion-dependent anemia and non-transfusion-dependent thalassemia (NTDT). We enrolled 243 patients with myelodysplastic syndromes (MDS), thalassemia major (TM), NTDT or other chronic anemia. Overall, 10% and 48% had cardiac and hepatic siderosis, respectively. Mean liver iron concentration (LIC) was above target range in all groups; mean myocardial T2^∗^ was normal. Hepatic siderosis was more prevalent than myocardial siderosis in patients with MDS, occurring in 54.4% and 4.4% of patients, respectively. As also observed in patients with NTDT or other anemia, hepatic siderosis was present in a large proportion of MDS patients who were chelation naïve (57.7%), as well as in patients receiving iron chelation therapy (ICT) (52.4%), despite a lower transfusion load compared with TM. Correlation between LIC and serum ferritin was observed across diseases; however, not all patients requiring ICT could be identified with serum ferritin alone, as serum ferritin underestimated LIC in 4.4% and overestimated LIC in 7.5% of patients. Exploratory analyses showed serum ferritin thresholds for liver siderosis detected by MRI at approximately 300 ng/mL higher in MDS than in TM. Most patients reported low–medium adherence to ICT; MRI assessment led to change in ICT in 46% of evaluable patients, including 52% of MDS patients. Accurate organ iron monitoring by MRI facilitated appropriate initiation of chelation, dose optimization and clinical decision making.

**Trial registration:** ClinicalTrials.gov: NCT01736540.

## Abstract 2

Supplemental Digital Content is available in the text

## Introduction

In patients receiving red blood cell (RBC) transfusions for transfusion-dependent anemia and patients with non-transfusion-dependent thalassemia (NTDT), iron overload is associated with significant morbidity and mortality.^1–3^ Regular monitoring of iron load is important to ensure prompt initiation and adjustment of iron chelation therapy (ICT) when appropriate. The benefits of ICT have been extensively studied in thalassemia major (TM) and are well established.^4–6^ However, in other transfusion-dependent anemia such as myelodysplastic syndromes (MDS) and other causes of chronic anemia (eg, myelofibrosis and leukemia), understanding of the impact of iron overload and ICT is heterogeneous. Chelation protocols, where available, are not consistently accepted or followed.^7–10^ Iron overload persists in some patients despite ICT, even in TM patients where the risks are widely accepted. This may be attributable to non-adherence,^11^ suboptimal dosing or inefficacy of ICT.

Serum ferritin is commonly used as an inexpensive, indirect measure of tissue iron and has been shown to correlate with liver iron concentration (LIC).^12–15^ There are, however, limitations in its use. Serum ferritin can be increased by factors that are independent of iron status (eg, inflammation, liver damage) and has been shown to have a weak correlation with myocardial T2^∗^ (mT2^∗^),^16^ an established magnetic resonance imaging (MRI) measurement of myocardial iron.^17–19^ The relationship between serum ferritin and organ iron load in diseases such as MDS has not been definitively shown, as there are many potential confounding variables. In patients on ICT, it has been demonstrated that up to half of patients with no serum ferritin response may be responding with respect to LIC, highlighting that serum ferritin should be interpreted with care.^20^ MRI is a non-invasive method, less affected by factors independent of tissue iron, that allows accurate, highly reproducible assessment of myocardial and hepatic iron.^21,22^ Increased availability of MRI has improved clinical outcomes in patients with TM,^23–25^ suggesting that it may also be beneficial in other diseases susceptible to iron overload.

The TIMES (Transfusional Iron overload assessed by Magnetic rESonance imaging) study is an epidemiological study that used MRI to assess the prevalence and severity of cardiac and hepatic siderosis in a large Australian population with diverse causes of transfusion-dependent anemia and NTDT, and evaluated the impact of MRI findings on treatment decisions. The impact of iron load and ICT on quality of life (QoL) was also assessed. The accuracy and effectiveness of serum ferritin as an indicator of organ iron load was evaluated across different disease groups.

## Materials and methods

### Eligibility criteria

Patients aged ≥18 years with a diagnosis of MDS (any International Prognostic Scoring System [IPSS] risk), TM, NTDT, or other causes of anemia, including sickle cell disease, Diamond–Blackfan anemia or myeloproliferative neoplasia, were enrolled. Patients were required to have a transfusion history of ≥20 packed RBC units and serum ferritin >500 ng/mL; patients with NTDT were required to have a serum ferritin level >300 ng/mL, with no minimum number of transfusions. Patients could have previously received ICT or be chelation naïve. Pregnant women, or patients with any condition preventing MRI, were not eligible.

### Study design

TIMES was a cross-sectional, epidemiological study designed to collect information on serum ferritin (the most commonly used parameter in clinical practice) and MRI assessment of iron levels, as well as associated clinical parameters, in a large cohort of patients at risk of iron overload due to chronic transfusions or NTDT. Serum ferritin was required to be assessed within 1 month before commencement of the study to determine eligibility. Patients attended 2 clinical visits ≤60 days apart. At the first visit, after informed consent, prospective data from physical examination and serum ferritin levels were collected. Patients also completed QoL questionnaires. At the second visit, MRI (FerriScan^®^) was performed to determine R2 LIC and mT2^∗^. Retrospective clinical data were collected, including hematologic parameters, treatment for anemia, medications, illnesses, serious adverse events (AEs) during the 12 months before enrollment, and ICT during the preceding 24 months. AEs and serious AEs were also recorded from the date of consent until the MRI was performed.

The study was conducted in accordance with the *Declaration of Helsinki* and an independent ethics committee for each study site approved the study protocol (ClinicalTrials.gov: NCT01736540).

### Assessments and statistical analysis

#### MRI assessment of liver and myocardial iron

The primary objective was to determine the prevalence and severity of liver and myocardial iron overload using MRI (R2 by FerriScan^®^ and mT2^∗^, respectively). MRI was performed within 60 days of consent. Hepatic siderosis was indicated by R2 LIC ≥7 mg Fe/g dry weight (dw); ≥5 mg Fe/g dw for NTDT.^13,26^ Myocardial siderosis was indicated by mT2^∗^ < 20 ms. Standard descriptive analyses were calculated for each disease group and according to ICT status: chelated (≥1 month's chelation in lifetime), minimally chelated (<1 month's chelation in lifetime), or chelation naïve.

#### Accuracy of serum ferritin in the assessment of organ iron load

The relationship between serum ferritin and LIC across the four disease groups was assessed by linear regression analysis on serum ferritin at screening and LIC measurements within 60 days of screening.

To determine how often serum ferritin underestimates liver iron load, the percentage of patients with serum ferritin levels in the clinically acceptable range (<1000 ng/mL for TM, MDS and other anemia, and <800 ng/mL for NTDT) but LIC above target range (≥7 mg Fe/g dw; NTDT ≥5 mg Fe/g dw) was calculated. Conversely, to assess how often serum ferritin overestimates LIC, serum ferritin levels >2000 ng/mL, that is, 2 times the chelation threshold in TM, MDS, and other anemia, and the frequency at which this was associated with LIC within the target range (ie, <7 mg Fe/g dw) was calculated. For NTDT, the same analysis was performed using serum ferritin >1600 ng/mL, that is, 2 times the chelation threshold of 800 ng/mL, and a target LIC of <5 mg Fe/g dw. These serum ferritin levels were chosen to ensure that patients with unambiguously elevated serum ferritin were identified, as the consequences of underestimating organ iron overload are of much greater clinical impact than overestimation. The frequencies are, therefore, not definitive, but provide a general assessment of the prevalence of overestimation.

#### Exploratory analysis to compare the threshold ranges of serum ferritin indicating liver siderosis in MDS, TM, and ‘other anemia’

Patients were classified into 2 groups according to LIC: ≥7 mg Fe/g dw (liver siderosis) and <7 mg Fe/g dw (no liver siderosis). To explore an appropriate threshold of serum ferritin to predict liver siderosis, a receiver operating characteristics (ROC) curve was produced for patients in MDS, TM and ‘other anemia’ groups; NTDT was not analyzed because of small patient numbers. A series of classification tables were produced with 100 ng/mL increments of serum ferritin as thresholds. At each serum ferritin level, all patients were classified as ‘positive based on serum ferritin’ if the value was greater than the threshold, and ‘negative based on serum ferritin’ if the value was less than or equal to the threshold. A cross-tabulation of serum ferritin result (positive or negative) and the result based on LIC (positive or negative) was produced (see SDC, Table 1, Supplemental Digital Content). True-positive and false-positive rates were calculated at each serum ferritin threshold and used to construct a ROC curve. A logistic regression model was fitted of the form: logit(p(liver siderosis)) = serum ferritin value in ng/mL and the c-statistic used to provide an estimate of the area under the curve (AUC). If serum ferritin is not useful as a predictor of liver siderosis, AUC is 0.5. The maximum AUC possible (for a perfect test) is 1.0. The observed AUC was tested against a null model (AUC 0.5) to determine whether serum ferritin provides a statistically significant improvement in prediction of liver siderosis over chance alone. To explore the potential serum ferritin threshold to use as the cut-off value for indicating liver siderosis, the ROC curves and classification tables at each serum ferritin threshold were examined. In addition, the optimal threshold was calculated as the point on the ROC curve closest to (0,1).^27^ This point can be found as: 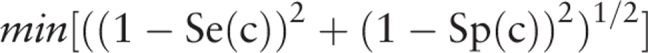


where min is ‘the minimum of’, Se is sensitivity, Sp is specificity (true-negative rate, ie, 1–Sp is the false-positive rate) and c is the threshold.

#### Adherence, quality of life, and the impact of MRI results on treatment decisions

At screening, patients receiving ICT completed the medication adherence questionnaire based on the Medication Adherence Rating Scale (MARS) questionnaire.^28^ Three additional questions from questionnaires used in studies assessing ICT adherence were also adapted and included (see SDC, Table 2, Supplemental Digital Content).^11,29,30^ Questions were constructed to avoid a yes-saying bias, coded as 1 = Yes and 0 = No, and combined to calculate an adherence score (0–6). A score of >2 indicates low adherence, 1 or 2 medium, and 0 high adherence. If fewer than 4 questions were answered, the adherence score was not calculated. QoL was assessed using the Short Form 36^®^ (SF-36^®^) Health Survey.^31^

The level of adherence and its effect on organ iron load, and a possible effect of iron load or ICT on QoL, were assessed. Relative risk of siderosis was calculated using a generalized linear-model exploratory analysis with patient adherence score as the outcome variable and treatment status, hepatic and myocardial siderosis as predictors. Generalized linear-model exploratory analysis was employed to determine predictors of QoL, with QoL score as the outcome variable and disease status, chelation regimen, serum ferritin, mT2^∗^ and LIC as predictors.

After evaluation of MRI results, investigator treatment decisions regarding ICT were assessed using a questionnaire to determine whether the MRI result led to a change in ICT and, if so, which of the three measurements (serum ferritin, LIC or mT2^∗^) was the main determinant.

## Results

### Patient demographics, transfusion, and iron chelation history

In total, 243 patients were enrolled between February 2013 and May 2015 at 13 sites. Patients had MDS (n = 73, 30.0%; IPSS Low risk n = 35, Intermediate [Int]-1 n = 26, Int-2 n = 7, High n = 5), TM (n = 81, 33.3%), NTDT (n = 20, 8.2%), or other anemia (n = 69, 28.4%; Table 1). Patients with other anemia included myeloproliferative neoplasia (n = 16), acute myeloid leukemia (n = 14), acute lymphoblastic leukemia (n = 9), sickle cell disease (n = 9), aplastic anemia (n = 8), autoimmune hemolytic anemia (n = 3), Diamond–Blackfan anemia (n = 2), pure red cell aplasia (n = 2), chronic myeloid leukemia (n = 1), multifactorial (n = 1; autoimmune hemolysis, chronic infection, dysplasia), myeloma (n = 1), non-Hodgkin lymphoma (n = 1), pyruvate kinase deficiency (n = 1), and T-cell large granular lymphocytic leukemia (n = 1).

**Table 1:**
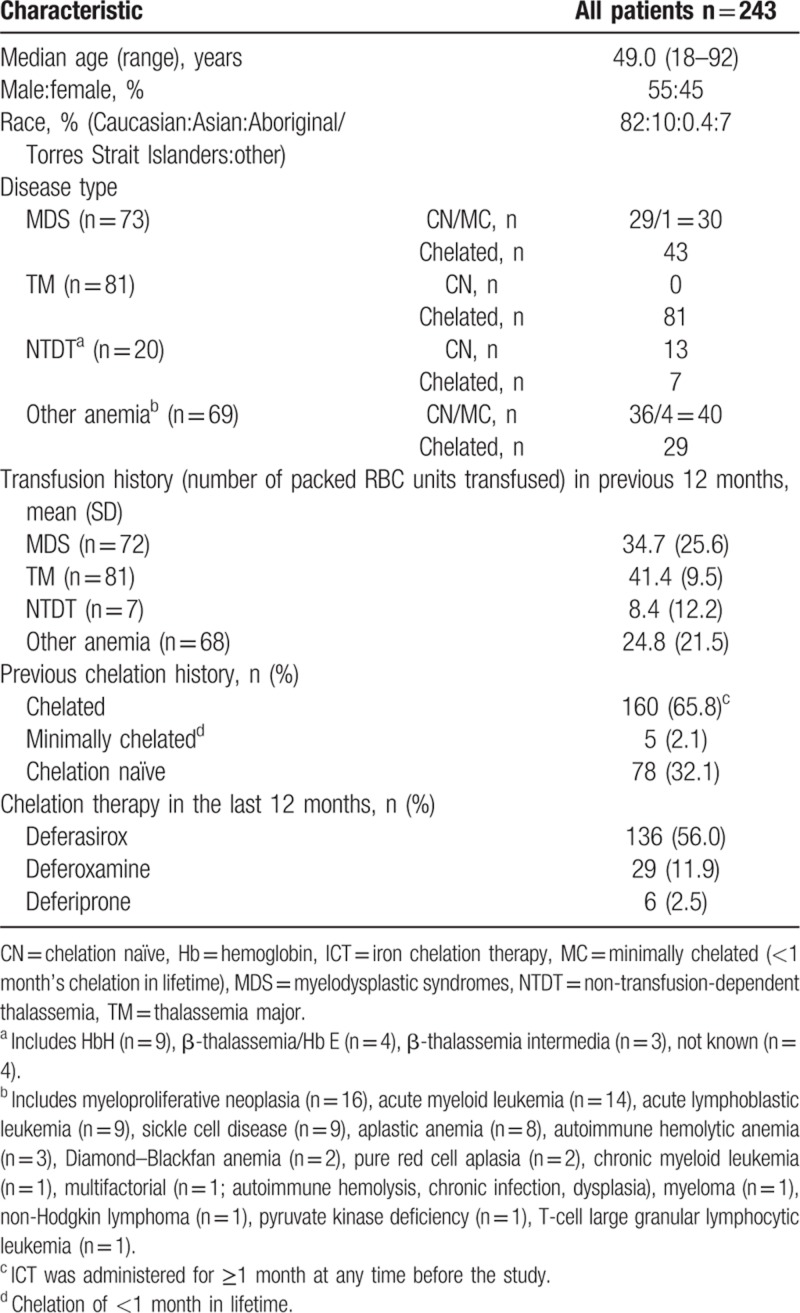
Patient characteristics, transfusion, and iron chelation history.

The mean number of packed RBC units (259 ± 16 mL per unit; hematocrit 0.58 ± 0.03) transfused in the 12 months prior to enrollment was 33.3 ± 21.0 (mean ± SD) units; in the different diseases, this was for MDS 34.7 ± 25.6 units, TM 41.3 ± 9.5 units, NTDT 8.4 ± 12.2 units and other anemia 24.8 ± 21.5 units. The mean lifetime duration of transfusion was longer for TM patients (who are transfused from childhood) at 29.8 ± 14.3 years, compared with MDS at 2.9 ± 2.6 years, NTDT at 10.2 ± 11.9 years, and other anemia at 3.1 ± 5.0 years. Patients did not necessarily receive ICT during the 60-day study period, but may have received it in the 24 months before consent; 65.8% of patients received ICT for ≥1 month at any time in the retrospective period. Of those patients who received ICT in the previous 12 months (n = 160), most (85.0%) received deferasirox (n = 136 [56.0% of the total population]), 18.1% (n = 29) received deferoxamine and 3.8% (n = 6) deferiprone; patients may have received more than 1 ICT agent. Five patients received minimal chelation (<1 month): one and four patients in the MDS and ‘other anemia’ groups, respectively. Due to these small numbers, results for chelation-naïve and minimally chelated patients are presented together, referred to as chelation naïve. Overall, 228 patients completed the study; 15 patients discontinued: unable to make MRI appointment (n = 6), withdrawal of consent (n = 3), not completing MRI within 45 days (n = 2), abnormal test result, protocol violation, death, intolerance of MRI (all n = 1).

### Prevalence and severity of myocardial and hepatic siderosis

Iron load measured by LIC, mT2^∗^ and serum ferritin is shown in Table 2. Target ranges for ICT for serum ferritin and LIC were established at <1000 ng/mL and 3 to 7 mg Fe/g dw, respectively, for TM,^13,32,33^ and this has been applied to the other transfusion-dependent diseases. For NTDT, previous analyses had indicated a serum ferritin target of <800 ng/mL and LIC ≤5 mg Fe/g dw.^26,34^ Mean LIC and serum ferritin were above the target range in all disease groups and remained above the target range in patients receiving ICT. Mean mT2^∗^ was normal (>20 ms) in all disease groups.

**Table 2:**
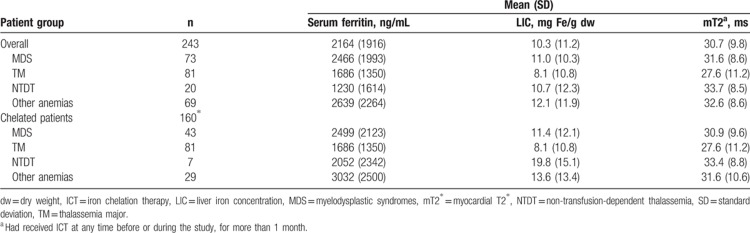
Measurements of iron load in all patients and in chelated patients.

**Figure 1:**
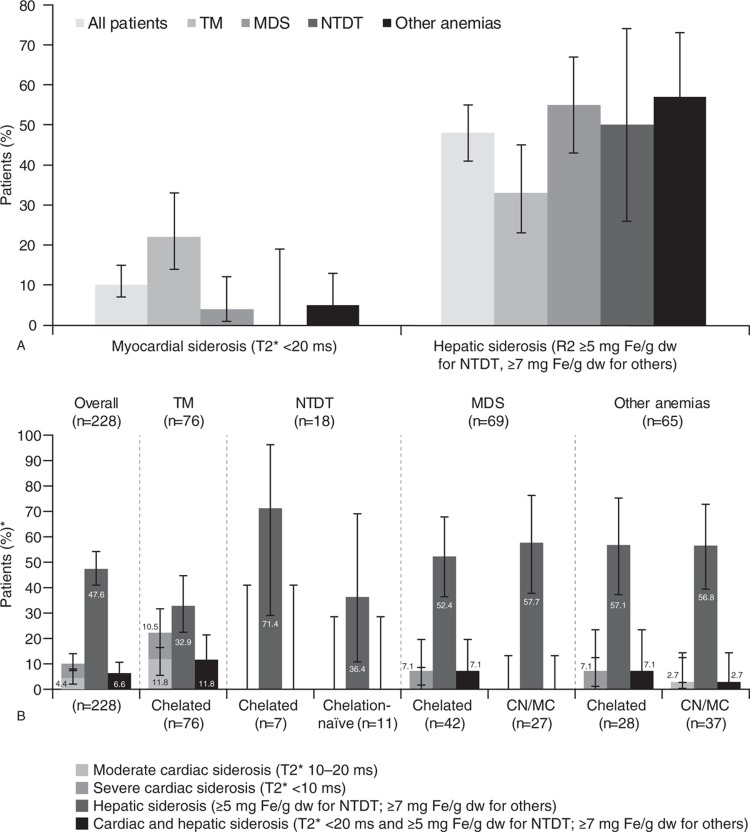
(A) Prevalence of iron overload across diseases and (B) prevalence and severity of iron overload in MDS, TM, NTDT, and other anemia. ^∗^Not all patients in the full analysis set had MRI data for assessment; bars show 95% confidence intervals. CN = chelation naïve, dw = dry weight, MC = minimally chelated, MDS = myelodysplastic syndromes, MRI = magnetic resonance imaging, NTDT = non-transfusion-dependent thalassemia, TM = thalassemia major.

The prevalence and severity of myocardial and hepatic siderosis varied across the disease groups and ICT status. Myocardial and hepatic siderosis were detected in 10% and 48% of all patients, respectively (Fig. 1A). For MDS, hepatic siderosis was more prevalent than myocardial siderosis, occurring in 54.4% and 4.4%, respectively. The prevalence of hepatic siderosis was similar in MDS (54.4%), NTDT (50.0%) and other anemia (56.9%), but higher than in TM (32.9%). The highest prevalence of myocardial siderosis was observed in TM, occurring in 22% of patients. For MDS, NTDT and other anemia, a sizeable proportion of chelation-naïve patients had hepatic siderosis: 57.7% in MDS, 36.4% in NTDT, 56.8% in other anemia (Fig. 1B). Even patients who had received ICT had a high prevalence of hepatic siderosis – over 50% of patients with MDS and other anemia, and 71.4% of NTDT patients.

In over half of all patients with myocardial siderosis (n = 13/23), the level of iron overload was severe (mT2^∗^ <10 ms; Fig. 1B). Of those patients receiving ICT, myocardial siderosis was severe in all 3 MDS patients who had abnormal mT2^∗^ (3/42; 7.1%; Fig. 1B). For TM, moderate myocardial siderosis was seen in 9 patients (mT2^∗^ 10–20 ms; 11.8%) and severe siderosis in 8 patients (10.5%; Fig. 1B) despite ICT. Two patients with other anemia (7.1%) who had received ICT had severe myocardial siderosis, while 1 (2.7%) chelation-naïve patient had moderate myocardial siderosis (Fig. 1B). No NTDT patient had myocardial siderosis.

### Relationship between LIC and serum ferritin

**Figure 2:**
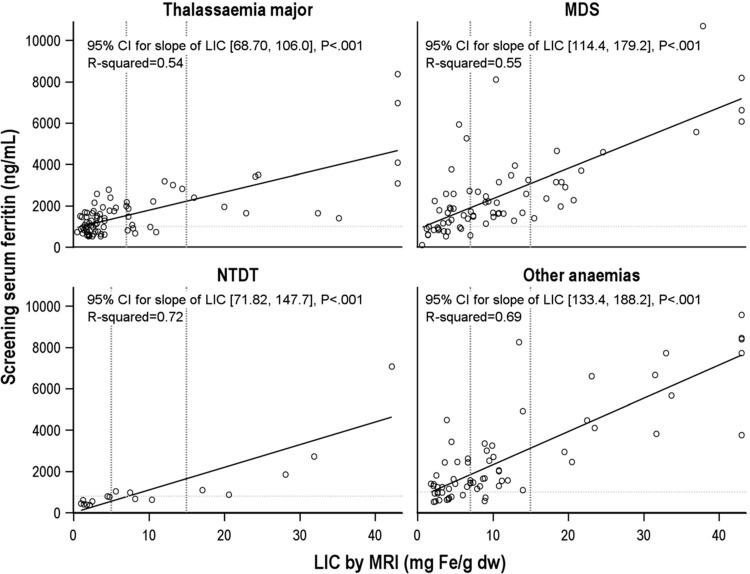
**Correlation between liver iron concentration and serum ferritin in the 4 disease groups**. For disease types other than NTDT, dashed reference lines indicating target levels for iron chelation are shown at 1000 ng/mL for serum ferritin, and 7 mg and 15 mg Fe/g dw for LIC; for NTDT, dashed reference lines are shown at 800 ng/mL for serum ferritin, and at 5 and 15 mg Fe/g dw for LIC. CI = confidence interval, dw = dry weight, LIC = liver iron concentration, MDS = myelodysplastic syndromes, NTDT = non-transfusion-dependent thalassemia, SD = standard deviation.

Regression analysis demonstrated a positive correlation between LIC and serum ferritin in all diseases (p < 0.001; Fig. 2), confirming the utility of serum ferritin levels >1000 ng/mL as an accurate clinical indicator of liver iron overload in most patients. However, 4.4% (n = 10/227) had high LIC (≥7 mg Fe/g dw [≥5 mg Fe/g dw for NTDT]) and relatively low serum ferritin (≤1000 ng/mL [≤800 ng/mL for NTDT]); in the 4 disease groups, the percentages were 1.4% (MDS), 11.8% (NTDT), 6.6% (TM) and 3.1% (other anemia).

Conversely, 7.5% of patients had highly elevated serum ferritin (>2000 ng/mL for MDS, TM, and other anemia; >1600 ng/mL for NTDT) but LIC within the target range (MDS 10.1%, TM 5.3%, other anemia 9.2%; NTDT nil), that is, approximately 1 in 10 MDS patients with highly elevated serum ferritin did not have increased liver iron.

**Figure 3:**
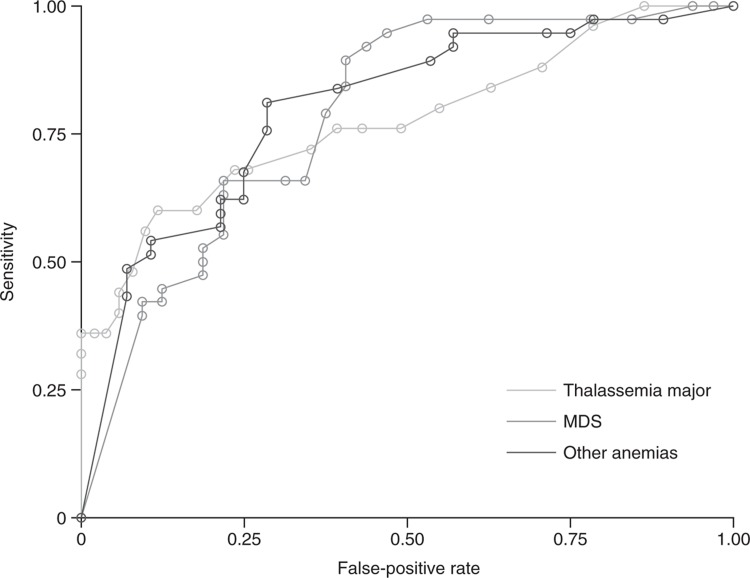
**ROC analysis of liver iron concentration against serum ferritin**. ROC analysis of LIC against serum ferritin values in MDS (n = 70, AUC 0.783, p < 0.001), TM (n = 76, AUC 0.779, p < 0.001), and other anemia (n = 65, AUC 0.808, p < 0.001). LIC ≥7 mg Fe/g dw denotes liver siderosis in all disease groups. For each line, markers going from right to left represent serum ferritin threshold values from 0 to 3000 ng/mL in steps of 100 ng/mL. Note that some values of serum ferritin may have the same sensitivity and false-positive rate (see data in SDC, Table 1, Supplemental Digital Content). AUC = area under the curve, dw = dry weight, LIC = liver iron concentration, MDS = myelodysplastic syndromes, TM = thalassemia major.

ROC curves were constructed to compare levels of serum ferritin indicating the presence of liver siderosis in MDS, TM, and other anemia; NTDT was not analyzed due to small numbers (Fig. 3). These are derived from the true- and false-positive rates for each serum ferritin threshold in predicting liver siderosis, measured by MRI LIC (SDC, Table 1, Supplemental Digital Content). Owing to the relatively small patient numbers in each disease group (MDS n = 70; TM n = 76; other anemia n = 65), this analysis is considered exploratory. For TM, for which most data on correlations between serum ferritin and LIC have previously been derived,^13^ our data showed that a threshold of 1400 ng/mL provided 72% sensitivity but a 35% false-positive rate, while raising the cut-off to 1500 ng/mL reduced the sensitivity to 68% but decreased the false-positive rate to 24%. In contrast, for MDS, a threshold of 1500 ng/mL was too low, as the false-positive rate was 41% despite higher sensitivity at 84%. A cut-off of 1700 ng/mL would be more clinically appropriate, with a false-positive rate of 34%, although sensitivity was reduced to 66%. For other anemia, a threshold of 1500 ng/mL provided sensitivity of 76% and false-positive rate of 29%, similar to TM. The results from the equation-based assessment of the optimal threshold were similar, indicating thresholds of 1900 ng/mL for MDS (sensitivity 66%, false positive 22%), 1600 ng/mL for TM (sensitivity 68%, false positive 24%) and 1400 ng/mL for other anemia (sensitivity 81%, false positive 29%).

### Patient adherence, impact on iron overload and QoL

**Figure 4:**
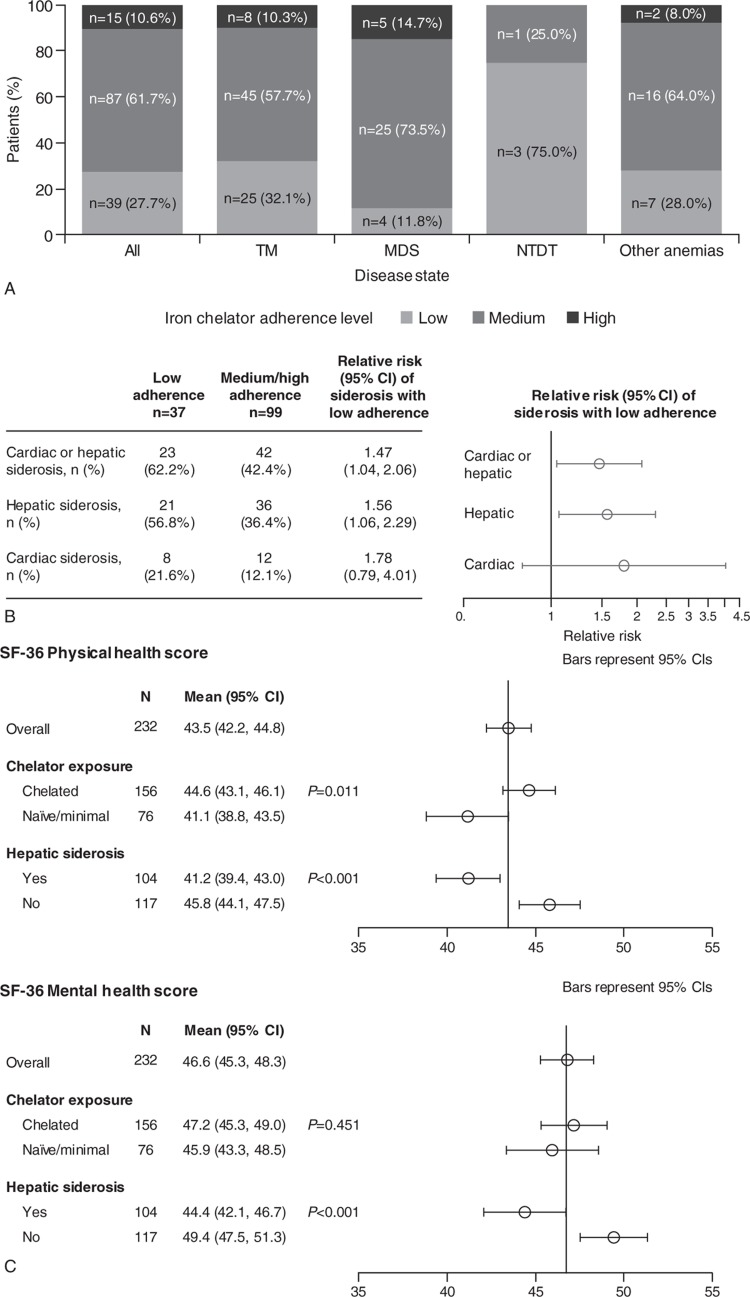
(A) Patient adherence to chelation therapy across diseases, (B) the impact of adherence on iron overload, the risk of siderosis with low adherence, and (C) the association between siderosis and iron chelation therapy on patient quality of life. SF-36 scores are measured on a scale of 0 to 100; higher scores indicate better QoL. CI = confidence interval, MDS = myelodysplastic syndromes, NTDT = non-transfusion-dependent thalassemia, QoL = quality of life, SF-36^®^ = 36-item Short Form Health Survey, TM = thalassemia major.

Overall, 89.4% of all patients receiving ICT reported medium-to-low adherence (MDS, 85.3%; TM, 89.8%; NTDT, 100%; other anemia, 92.0%; Fig. 4A). Low adherence was a risk factor for both myocardial and hepatic siderosis (Fig. 4B), with a relative risk of 1.56 (95% confidence interval [CI] 1.06, 2.29) for hepatic and 1.78 (95% CI 0.79, 4.01) for myocardial siderosis.

Hepatic siderosis had a negative impact on patient QoL (Fig. 4C; mean SF-36^®^ physical health score 41.2, 95% CI 39.4, 43.0; p < 0.001; mean SF-36^®^ mental health score 44.4, 95% CI 42.1, 46.7; p < 0.001) compared with no hepatic siderosis (mean physical health score 45.8, 95% CI 44.1, 47.5; mean mental health score 49.4, 95% CI 47.5, 51.3). By contrast, ICT for >1 month was associated with better QoL (mean physical health score 44.6, 95% CI 43.1, 46.1; p = 0.011) compared with chelation-naïve patients (41.1, 95% CI 38.8, 43.5; Fig. 4C).

### The impact of MRI assessment on clinical management

MRI assessment led to a change in chelation in 105 (45.9%) of all evaluable patients (n = 229), including 52% (36/69) of MDS patients, and in ∼60% of chelation-naïve/minimally chelated patients with MDS (17/27 [63.0%]) and other anemia (22/37 [59.5%]; SDC, Table 3, Supplemental Digital Content). In patients who had received ICT for >1 month, the most frequent change was increasing chelator dose (MDS, 11/16 [68.8%]; TM, 20/23 [87.0%]; NTDT, 4/7 [57.1%]; other anemia, 5/6 [83.3%]). In chelation-naïve/minimally chelated patients, (re-)starting ICT was the predominant change (20/23; 87.0%). LIC was the main driver for the investigators’ decisions regarding ICT in MDS (42.0%), NTDT (44.4%), and other anemia (56.3%; SDC, Fig. 1, Supplemental Digital Content), whereas mT2^∗^ was the main determinant in TM (63.8%).

### Safety, including incidence of infections and relationship with LIC

During the study period (≤60 days), 24 patients experienced 86 AEs (MDS n = 43; TM, n = 10; other anemia n = 33), 34 severe and life-threatening AEs (MDS n = 12; TM, n = 5; other anemia n = 17), and 29 serious AEs (MDS n = 11; TM, n = 5; other anemia n = 13). There were no withdrawals due to AEs. The most common AEs (≥3 patients) were infection and infestations (n = 13), anemia (n = 5), febrile neutropenia (n = 3), abdominal pain (n = 3), diarrhea (n = 3), fatigue (n = 3) and headache (n = 3). Of the infections and infestations, the 13 events occurred in 9 patients – MDS (n = 1), TM (n = 5), other anemia (n = 3). Of these, hepatic siderosis (as a measure of total body iron) was found in 1/1 MDS patient, 2/5 TM and 2/3 ‘other anemia’ patients, that is, infections were reported in 5/9 patients with hepatic siderosis and 4/9 with LIC within target range.

## Discussion

The TIMES study provides a real-life assessment of the magnitude of iron load and insight into the impact of MRI on treatment decisions in a large population of patients with transfusion-dependent anemia or NTDT. This population is representative of many communities where ICT has been available to patients with both inherited and acquired chronic anemia over a long time. Despite this, we demonstrate a high prevalence of hepatic siderosis in all disease groups (>50% in MDS, NTDT, and other anemia, and 33% in TM) and show that nearly a quarter of TM patients had myocardial siderosis despite ICT. MRI analysis resulted in a change in management in nearly half of all evaluable patients. Patients in all disease groups reported low-to-medium adherence to ICT (despite the majority receiving oral therapy), shown to be a risk factor for both hepatic and cardiac siderosis. A clear relationship was demonstrated between QoL and hepatic siderosis, and with ICT. A significant proportion of patients with MDS and other anemia who were chelation naïve or minimally chelated had hepatic siderosis. While there are significant geographical variations, in the routine practice of this jurisdiction, MRI analysis is mainly performed in metropolitan centers for patients with hemoglobinopathies, but rarely in MDS and other anemia.

While there were no marked differences in transfusion requirements between the transfusion-dependent disease groups (MDS, TM, other anemia) in the 12 months prior to the study (nor in the previous 48 months – results collected but not presented), the mean lifetime duration of transfusion was significantly shorter for MDS (2.9 years) and ‘other anemia’ (3.1 years) compared with 29.8 years in TM. Despite this, the prevalence of hepatic siderosis was >50% in MDS and ‘other anemia’, emphasizing the importance of the finding of high prevalence of hepatic iron overload in these 2 groups. Our study indicates that despite the markedly lower mean duration of transfusion in MDS patients, the prevalence of hepatic siderosis (54.4%) was higher than in TM patients (32.9%). While myocardial siderosis was much less prevalent in MDS (4.4%) and other anemia (4.6%) than in TM (22%), it is possible that with increased years of transfusion, the prevalence may rise. However, MDS being a myeloid neoplasm, the likelihood of continuous transfusion for a prolonged duration for MDS patients is obviously lower than in TM.

Maintaining serum ferritin <1000 ng/mL has been a key target of ICT in clinical practice and, as demonstrated in TM, is associated with higher rates of survival and improved organ function.^17,35^ This study demonstrated good correlation between serum ferritin and LIC in most patients and across all diseases, confirming the utility of serum ferritin as a marker of iron overload. However, it was also clear that not all patients in need of ICT can be identified using serum ferritin, as some patients with high LIC had relatively low serum ferritin (4.4% in this study). Conversely, some patients with high serum ferritin may have LIC within the target range (overall 7.5%; approximately 10% in MDS and other anemia, and 5% in TM). There was no overestimation of LIC by serum ferritin in NTDT, consistent with previous findings in NTDT that serum ferritin often underestimates liver iron load.^18,36,37^ In this study, serum ferritin levels from single time points were used for all analyses. While mean serum ferritin values over time may provide a more accurate picture for individual patients, this would not be consistent with the epidemiological, cross-sectional study design. The good correlation shown between LIC and serum ferritin in a relatively large number of patients, confirming previous findings, supports the integrity of the methodology.

In those patients in whom serum ferritin was not an optimal marker for iron overload, MRI would be a suitable alternative as it provides accurate and reproducible assessment of liver and myocardial iron levels.^16,21,22^ However, in some countries, cost may be prohibitive for its widespread use. In MDS, our exploratory ROC analysis demonstrated that the serum ferritin threshold for hepatic siderosis was 1700 to 1900 ng/mL, compared with that of TM of 1500 to 1600 ng/mL, for which a chelation threshold of 1000 ng/mL has been adopted in TM guidelines. This observation would suggest that a chelation threshold of approximately 300 ng/mL higher in MDS would be appropriate. The cause of this difference is not clear but could reflect different molecular pathways and mechanisms of iron load in the underlying diseases,^38–40^ or the increased effect of an inflammatory or stress state in MDS.^41^ However, as the threshold ranges were found to be similar among the disease groups, it would be reasonable and practical to use the same chelation threshold in TM and MDS, bearing in mind the higher false-positive rate in MDS of 30% to 35% in this study. Moreover, using 2 times the chelation threshold as a marker of an unambiguously elevated serum ferritin, we showed that serum ferritin erroneously overestimated LIC to be above target range in 1 in 10 MDS patients. On the basis of the threshold for liver siderosis in MDS estimated at 1700 to 1900 ng/mL, we propose that MRI assessment of LIC would be particularly useful in MDS patients with serum ferritin of 1500 to 2000 ng/mL. It should be noted that the serum ferritin thresholds in this study were determined in a heterogeneous group of patients with respect to ICT. Different cut-offs might be derived in a clinical setting where all patients are receiving chelation. The timing of ICT commencement in relation to the measurement of serum ferritin may also be a factor, as serum ferritin would likely ‘respond’ more quickly to ICT than organ iron.

There are other potential biomarkers of iron overload such as non-transferrin-bound iron (NTBI) and labile plasma iron (LPI),^42^ N-terminal brain natriuretic peptide (NT-BNP)^43^ and hepcidin,^44^ but data are still limited and not yet conclusive. Despite their potential utility, techniques for measuring NTBI and LPI are either technically demanding or not standardized, while LPI can vary dramatically in response to extraneous factors, such as fever or the timing of chelator dosing.^45^ Hepcidin levels are affected by multiple factors such as ineffective erythropoiesis, recent transfusions, inflammation, liver disease and renal dysfunction, and are less likely than MRI to reflect iron stores alone.^44^ NT-BNP has been found to correlate with cardiac siderosis, but data are also limited.^43^ These parameters are, therefore, still considered research tools and, unlike serum ferritin, have not been incorporated into routine practice.

Although many studies have demonstrated the efficacy of ICT in reducing iron overload, there was a high prevalence of hepatic and cardiac siderosis in patients receiving ICT in the current study. Poor adherence was confirmed as a cause in this study, with relative risks of 1.56 and 1.78, respectively, for myocardial and hepatic siderosis. Detection and monitoring of iron overload, ideally with MRI, are also important in establishing an effective ICT regimen,^26,32,33^ as demonstrated in this study, where more than 50% of chelation-naïve MDS patients had evidence of liver siderosis, and change in ICT occurred in nearly half of all patients due to MRI assessment. Nearly all changes were to increase chelator dose or initiate ICT, suggesting that patients had been receiving suboptimal doses, or the level of iron overload had not been fully appreciated from interpretation of serum ferritin. Thus, suboptimal diagnosis, monitoring and chelator dosing in addition to poor adherence are all factors shown in this study for the high prevalence of siderosis. Other possible causes not assessed in this study include the timing of ICT initiation in relation to the start of transfusion, as early use has been associated with improved survival in the hemoglobinopathies.^46^ These data highlight the importance of tailoring ICT dose and regimen on an individual basis according to MRI results. MRI has become the gold standard for measurement of iron load because it provides accurate and reproducible results across multiple organs and is non-invasive;^47^ however, when resources are limited, careful consideration is required to identify those at most risk and decide which patients to screen with MRI.^47^ Our data on serum ferritin thresholds for hepatic siderosis have provided guidance especially in MDS patients, indicating that those with serum ferritin of 1500 to 2000 ng/mL would particularly benefit from MRI assessment. This strategy would be especially useful in MDS patients with acute-phase reactions, such as recurrent infection, or ongoing inflammation due to rheumatological diseases or therapy-related myeloid neoplasms. In addition, only 1.4% of MDS patients with serum ferritin <1000 ng/mL had elevated LIC, indicating that MRI in this group would have a low yield and would not be recommended, especially where resources are limited.

There are limited data on the incidence and consequences of iron overload in MDS and other hematologic disorders. European MDS and acute myeloid leukemia registry data demonstrated the prognostic importance of transfusion dependence for overall survival,^48^ while more recent Australian and European MDS registry data also demonstrated a poor prognostic impact of transfusion dependence, independent of revised IPSS.^49^ Although a link between iron overload and progression of MDS has not been established, preclinical studies have suggested that iron overload causes DNA damage that may contribute to leukemic transformation,^50,51^ and there are case reports of improvement in transfusion dependence in MDS patients treated by ICT.^52,53^ As such, improvement of iron control through the use of MRI could potentially lead not only to improved organ function but a reduction in disease transformation and transfusion requirements in MDS. Furthermore, many patients in the ‘other anemia’ group in the community are not routinely monitored for iron overload, nor would they receive ICT. In this study, MRI assessment has highlighted a large number of patients with MDS and other anemia (>50%) to have hepatic siderosis, most of whom were chelation naïve. The clinical effects of this untreated iron load are worthy of further investigation. Furthermore, although only a small number of patients (6/134) with MDS and other anemia had myocardial siderosis, the level was moderate to severe.

This study has confirmed previous observations that myocardial siderosis is more prevalent in TM than MDS, NTDT and other anemia. Different mechanisms in myocardial iron uptake and metabolism have been proposed for this finding.^54^ Differences in hepcidin levels may also play a role, being most profoundly suppressed in NTDT, less so in TM (regular transfusion suppresses ineffective erythropoiesis and erythroferrone, which inhibits hepcidin production),^55,56^ normal or elevated in sickle cell disease^57,58^ and reported as elevated in MDS in one study.^57^ Elevated hepcidin inhibits iron release from the reticuloendothelial system, reducing iron accumulation in parenchymal cells. Differences in the effect of hepcidin/ferroportin in the heart and liver may partly account for differential organ iron accumulation among the diseases.^54,57^

This study clearly demonstrated that hepatic siderosis is associated with reduced QoL, and that patients who had received ICT experienced better QoL than chelation-naïve patients. Although it is not possible to determine the impact of the underlying disease or comorbidities on QoL, most (84%) of the MDS patients had IPSS Low or Int-1 risk. It is, therefore, less likely that the underlying disease is the primary cause of the QoL differences.

Given the known, increased predisposition to infection in patients with iron overload, and the possible impact of infection on serum ferritin, we reviewed whether a relationship between iron overload and infection was evident in our cohort. Thirteen infections in 9 patients were reported during the study; of these patients, 5/9 had hepatic siderosis. While the low incidence of infections could be the result of under-reporting and the short study duration, our findings do not show evidence of hepatic iron overload conferring an increased risk of infections, in the context of the much larger number of patients with hepatic siderosis in whom no infections were reported.

The TIMES study provides a real-life assessment of myocardial and hepatic siderosis in a large population of patients with heterogeneous causes of chronic anemia receiving transfusions, and demonstrates that iron overload still occurs in a high proportion of susceptible patients, including those receiving ICT. Although serum ferritin values identified most patients with high LIC (only 4.4% not identified), a small but important number of patients would be missed using serum ferritin alone, while the availability of MRI led to an initiation or adjustment of ICT in almost half the patients. Serum ferritin thresholds indicating liver siderosis were 200 to 300 ng/mL higher in MDS (1700–1900 ng/mL) than in TM (1500–1600 ng/mL); thus, MRI LIC assessment in MDS patients with serum ferritin at 1500 to 2000 ng/mL would be particularly useful. Conversely, we showed that underestimation of hepatic iron by serum ferritin is rare in MDS (1.4%). Hence, MRI assessment in MDS patients with serum ferritin <1000 ng/mL would have a low yield and is unlikely to be beneficial. Overall, MRI monitoring in patients with transfusion-dependent anemia and NTDT provided a more accurate assessment of iron load, facilitating appropriate initiation of ICT, dose optimization and clinical decision making.

## Supplementary Material

Supplemental Digital Content
