# Supplementary material for: Cardiac and hepatic siderosis in myelodysplastic syndrome, thalassemia and diverse causes of transfusion-dependent anemia: the TIMES study
Source: Hemasphere. 2019 Jun 1;3(3):e224. doi: 10.1097/HS9.0000000000000224 (PMC6746020; doi:10.1097/HS9.0000000000000224)
Supplement: Supplemental Digital Content [file hs9-3-e224-s001.docx]

**Cardiac and hepatic siderosis in diverse causes of transfusion-dependent anemia and non-transfusion-dependent thalassemia: the TIMES study**

**SDC Table 1. True-positive and false-positive rates for detecting liver siderosis using serum ferritin threshold values in increments of 100 ng/mL, against the reference test of LIC ≥7 mg Fe/g dw**

|  | **MDS  (siderosis positive n=38, negative n=32)** | | **TM  (siderosis positive n=25, negative n=51)** | | **Other  (siderosis positive n=37, negative n=28)** | |
| --- | --- | --- | --- | --- | --- | --- |
| **Serum ferritin cut-off (ng/mL)** | **True positive (%)** | **False positive (%)** | **True positive (%)** | **False positive (%)** | **True positive (%)** | **False positive (%)** |
| 0 | 100.0 | 100.0 | 100.0 | 100.0 | 100.0 | 100.0 |
| 100 | 100.0 | 96.9 | 100.0 | 100.0 | 100.0 | 100.0 |
| 200 | 100.0 | 96.9 | 100.0 | 100.0 | 100.0 | 100.0 |
| 300 | 100.0 | 96.9 | 100.0 | 100.0 | 100.0 | 100.0 |
| 400 | 100.0 | 96.9 | 100.0 | 100.0 | 100.0 | 100.0 |
| 500 | 100.0 | 93.8 | 100.0 | 100.0 | 100.0 | 100.0 |
| 600 | 97.4 | 84.4 | 100.0 | 86.3 | 97.3 | 89.3 |
| 700 | 97.4 | 84.4 | 96.0 | 78.4 | 97.3 | 78.6 |
| 800 | 97.4 | 78.1 | 88.0 | 70.6 | 94.6 | 75.0 |
| 900 | 97.4 | 62.5 | 84.0 | 62.7 | 94.6 | 71.4 |
| 1000 | 97.4 | 53.1 | 80.0 | 54.9 | 94.6 | 57.1 |
| 1100 | 97.4 | 53.1 | 76.0 | 49.0 | 91.9 | 57.1 |
| 1200 | 94.7 | 46.9 | 76.0 | 43.1 | 89.2 | 53.6 |
| 1300 | 92.1 | 43.8 | 76.0 | 39.2 | 83.8 | 39.3 |
| 1400 | 89.5 | 40.6 | 72.0 | 35.3 | 81.1 | 28.6 |
| 1500 | 84.2 | 40.6 | 68.0 | 25.5 | 75.7 | 28.6 |
| 1600 | 78.9 | 37.5 | 68.0 | 23.5 | 67.6 | 25.0 |
| 1700 | 65.8 | 34.4 | 60.0 | 17.6 | 62.2 | 25.0 |
| 1800 | 65.8 | 31.3 | 60.0 | 11.8 | 62.2 | 21.4 |
| 1900 | 65.8 | 21.9 | 56.0 | 9.8 | 62.2 | 21.4 |
| 2000 | 63.2 | 21.9 | 48.0 | 7.8 | 59.5 | 21.4 |
| 2100 | 63.2 | 21.9 | 48.0 | 7.8 | 56.8 | 21.4 |
| 2200 | 55.3 | 21.9 | 44.0 | 5.9 | 56.8 | 21.4 |
| 2300 | 52.6 | 18.8 | 40.0 | 5.9 | 56.8 | 21.4 |
| 2400 | 50.0 | 18.8 | 36.0 | 3.9 | 56.8 | 21.4 |
| 2500 | 47.4 | 18.8 | 36.0 | 3.9 | 54.1 | 10.7 |
| 2600 | 44.7 | 12.5 | 36.0 | 2.0 | 51.4 | 10.7 |
| 2700 | 42.1 | 12.5 | 36.0 | 2.0 | 48.6 | 7.1 |
| 2800 | 42.1 | 9.4 | 36.0 | 0.0 | 48.6 | 7.1 |
| 2900 | 39.5 | 9.4 | 32.0 | 0.0 | 48.6 | 7.1 |
| 3000 | 39.5 | 9.4 | 28.0 | 0.0 | 43.2 | 7.1 |

dw, dry weight; LIC, liver iron concentration; MDS, myelodysplastic syndromes; TM, thalassemia major.

**SDC Table 2. Medical adherence questionnaire**

1. Do you ever forget to take your chelation therapy?
2. Are you careless at times about taking your chelation therapy?
3. When you feel better, do you sometimes stop taking your chelation therapy?
4. Sometimes if you feel worse when you take your chelation therapy, do you stop taking it?
5. I take my medication only when I am feeling sick
6. By staying on medication, I can prevent getting sick
7. In the past week, did you forget to take your chelation therapy on more than 1 day?
8. In general, in the last 4 weeks, did you follow the chelation therapy regimen exactly as directed by your doctor?
9. In general, in the last 4 weeks, did you think about stopping your chelation therapy?

**SDC Table 3. Summary of MRI-based treatment decisions**

| Change to ICT | Overall | MDS | | TM | NTDT | | Other anemias | |
| --- | --- | --- | --- | --- | --- | --- | --- | --- |
| Any change, n (%) | **n=229** | **CN/MC n=27** | **ICT n=42** | **ICT n=77** | **CN/MC n=11** | **ICT n=7** | **CN/MC n=37** | **ICT n=28** |
| Yes | 105 (45.9) | 17 (63.0) | 19 (45.2) | 26 (33.8) | 3 (27.3) | 7 (100.0) | 22 (59.5) | 11 (39.3) |
| No | 124 (54.1) | 10 (37.0) | 23 (54.8) | 51 (66.2) | 8 (72.7) | 0 (0.0) | 15 (40.5) | 17 (60.7) |
| Type of change, n (%) | **n=75** | **CN/MC n=13** | **ICT n=16** | **ICT n=23** | **CN/MC n=2** | **ICT n=7** | **CN/MC n=8** | **ICT n=6** |
| Start ICT therapy | 27 (36.0) | 10 (76.9) | 4 (25.0)* | 0 (0.0) | 2 (100.0) | 3 (42.9)* | 8 (100.0) | 0 (0.0) |
| Stop ICT therapy | 0 (0.0) | 0 (0.0) | 0 (0.0) | 0 (0.0) | 0 (0.0) | 0 (0.0) | 0 (0.0) | 0 (0.0) |
| Increase ICT dose | 43 (57.3) | 3 (23.1) | 11 (68.8) | 20 (87.0) | 0 (0.0) | 4 (57.1) | 0 (0.0) | 5 (83.3) |
| Decrease ICT dose | 2 (2.7) | 0 (0.0) | 1 (6.3) | 1 (4.3) | 0 (0.0) | 0 (0.0) | 0 (0.0) | 0 (0.0) |
| Switch to other ICT | 2 (2.7) | 0 (0.0) | 0 (0.0) | 1 (4.3) | 0 (0.0) | 0 (0.0) | 0 (0.0) | 1 (16.7) |
| Change ICT delivery | 1 (1.3) | 0 (0.0) | 0 (0.0) | 1 (4.3) | 0 (0.0) | 0 (0.0) | 0 (0.0) | 0 (0.0) |

Patient numbers based on evaluable patients; numbers for ‘type of change’ are lower than ‘any change – yes’ because some (n=30) specific responses were missing. *Patients with a history of receiving ICT restarted on therapy. CN, chelation naïve; ICT, iron chelation therapy; MC, minimally chelated; MDS, myelodysplastic syndromes; MRI, magnetic resonance imaging; NTDT, non-transfusion-dependent thalassemia; TM, thalassemia major.

**SDC Figure 1. Proportion of investigators (95% confidence interval) ranking LIC, mT2*, serum ferritin or other as having most influence on treatment decisions concerning iron chelation**


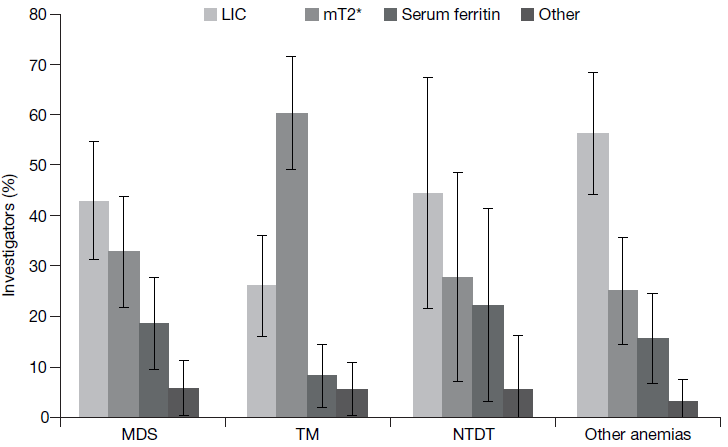


LIC, liver iron concentration; MDS, myelodysplastic syndromes; mT2*, myocardial T2*; NTDT, non­transfusion-dependent thalassemia; TM, thalassemia major.
